# Phytochemistry, Bioavailability, and Molecular Mechanisms Underlying Multitarget Anticancer Activity of *Aloe vera*

**DOI:** 10.3390/nu18122034

**Published:** 2026-06-22

**Authors:** Nimra Haroon, Adnan Amjad, Muhammad Maaz, Ahmad Mujtaba Noman, Nimra Anees, Zafarullah Muhammad, Mohibullah Shah, Waleed Al Abdulmonem

**Affiliations:** 1 Faculty of Food Science and Nutrition, Bahauddin Zakariya University, Multan 60800, Pakistan; nimraharoon48590@gmail.com (N.H.); ahmadmujtaba493@gmail.com (A.M.N.); nimraanees2002@gmail.com (N.A.); 2 College of Agriculture and Food Engineering, Baise University, Baise City 533000, China; zafwahla@bsuc.edu.cn; 3 Department of Biochemistry, Bahauddin Zakariya University, Multan 60800, Pakistan; mohib@bzu.edu.pk; 4 Department of Pathology, College of Medicine, Qassim University, Buraidah 51411, Saudi Arabia

**Keywords:** *Aloe vera*, anticancer activity, phytochemicals, Aloe-emodin, bioavailability, oxidative stress, molecular signaling pathways, natural therapeutics

## Abstract

**Background/Objectives**: Cancer, a multifactorial disease with uncontrolled cell growth, oxidative stress, inflammation, genomic instability, and molecular signaling pathways, is a global health concern, leading to the ~20 million newly diagnosed cases annually. Although conventional therapy has been shown to enhance the survival rates of cancer patients, its clinical efficacy is limited by certain side effects that occur as a result of treatment, thus necessitating the exploration of plant-derived bioactive compounds for their potential as safer and alternative supportive therapeutic agents. *Aloe vera*, known as the plant of immortality, comprises phytochemicals, such as anthraquinones (aloe-emodin, emodin, and aloin), polysaccharides (acemannan), flavonoids, and phenolic acids, which contribute to the pharmacological effect of the compound. **Methods**: This review summarizes the anticancer potential of *Aloe vera*, and the data were retrieved from databases, such as PubMed, Google Scholar, ScienceDirect, Web of Science, and Wiley Online Library, during the time period of 2015 to 2025. **Results**: The literature revealed that *Aloe vera* and its bioactive compounds have dose-dependent cytotoxic and anti-proliferative properties against hepatocellular, cervical, colorectal, lung, breast, prostate, and hematological cancers, which are significantly mediated by apoptosis and pyroptosis induction, reactive oxygen species (ROS) production, mitochondrial dysfunction, inhibition of angiogenesis and metastasis, and the modulation of key signaling pathways, particularly PI3K/Akt, MAPK, NF-кB, p53, and Wnt/β-catenin. Furthermore, anthraquinones, including Aloe-emodin, demonstrate potent anticancer effects at micro-molar doses, and polysaccharides increase immune reactions and provide tumor immunity. **Conclusions**: Conclusively, *Aloe vera* is a promising multi-target natural compound, particularly efficient in the treatment of cancer. However, despite significant therapeutic potential, more research on pharmacokinetics, standard dose, and controlled clinical trials of *Aloe vera* is required to validate clinical applicability.

## 1. Introduction

Nature has rewarded mankind with plants, facilitating the source of food, shelter, and medicine to improve the lifestyle and quality of life. Plants, especially medicinal plants, have been used since ancient civilization to prevent and treat various diseases [1]. The healthcare professionals, belonging to Ayurveda, Unani, Siddha, Traditional Chinese Medicine, Korean Traditional Medicine, Kampo, and Tibetan Medicinal systems, utilized the flowers, roots, fruits, petals, seeds, leaves, and stems, of these plants to prepare powder, decoction, extracts, juices, pastes, infusions, oils, syrups, pills, tablets, and capsules, which were used for the betterment of human health [2]. Although the advancement and modernization have developed more effective synthetic drugs, the rural communities (~70%) of developing countries still rely on medicinal plants as their primary source of treatment [3]. Moreover, the reported adverse health consequences have transformed one’s thinking and encouraged the development of safe and alternative drugs having target onset and better health outcomes [4]. In this regard, medicinal plants are safe and have multiple therapeutic activities, thereby contributing to the significance in scientific and commercial growth.

*Aloe vera*, traditionally known as “the plant of immortality”, is a miraculous plant of the genus Aloe, promptly grown in the warm, dry, tropical, and subtropical climates [5]. It is cultivated in Pakistan, India, China, Mexico, the United States, Thailand, the Philippines, Indonesia, South Africa, and Spain for its pharmaceutical and therapeutic attributes [6,7,8]. The unique phytochemicals, including anthraquinones and other phenolic compounds [9,10], are responsible for its remarkable pharmacological significance, such as dermatological [11,12], anti-inflammatory [13,14], antimicrobial [15], antioxidant [16], anticancer [17], analgesic [18], and laxative effects [19].

*Aloe vera* has gained valuable scientific attention for its potential role in oncology [20]. Cancer is an invasive, malignant, and multi-dimensional disease, which is characterized by uncontrolled proliferation of abnormal or modified cells, ultimately leading to tumor formation and progression [21,22]. It is proliferated among the individuals of both developed and developing countries, with an estimated 20-million global prevalence and ~9.7 million mortalities [23]. Moreover, the abnormal cells develop multiple characteristics, including insensitivity to anti-growth signals and apoptosis evasion, and consequently metastasize to the distant organs [24]. The management of cancer is a global concern, although various medications and therapies, such as chemotherapy, radiotherapy, targeted therapy, immunotherapy, hormone therapy, radiation therapy, surgery, bone marrow transplant, and gene therapy, are available, but their adverse effects, like hair loss, fatigue, nausea, vomiting, immune-related inflammation, need to be managed by other supportive therapies [25]. The emergence of plant-based therapeutic approaches has considerable importance due to their safe and effective outcomes [26]. The anticancer potential of *Aloe vera* has been supported by multiple studies, which is induced by promoting apoptosis, reducing inflammation, modulating immune responses, and inhibiting cancer cell proliferation [27]. However, the existing literature is scattered across experimental models and cancer types, impeding to develop a comprehensive understanding of its therapeutic approaches. Keeping in mind the recent scenario, this review was structured to focus on the in vivo and in vitro anticancer potential of *Aloe vera* against various cancers, such as breast, cervical, hepatocellular, prostate, lung, blood, and colorectal cancers. Moreover, phytochemicals, in addition to their associated mechanisms in cancer management, are the focus of this particular review.

## 2. Materials and Methods

This review was conducted by systematically searching relevant data from the online databases, such as Google Scholar, PubMed, Science Direct, Web of Science, and Wiley Online Library. To ensure reproducibility and to scrutinize relevant studies, following Boolean search combinations were used and adapted according to the search options of each database: (“*Aloe vera*” OR “Aloe barbadensis” OR “Aloe barbadensis Miller”) AND (“cancer” OR “tumor” OR “tumour” OR “carcinoma” OR “neoplasm”) AND (“anticancer” OR “antitumor” OR “anti-tumor” OR “cytotoxic” OR “antiproliferative” OR “anti-proliferative”); (“*Aloe vera*” OR “Aloe barbadensis”) AND (“phytochemicals” OR “bioactive compounds” OR “anthraquinones” OR “aloe-emodin” OR “emodin” OR “aloin” OR “acemannan” OR “polysaccharides” OR “flavonoids” OR “phenolic compounds”); (“*Aloe vera*” OR “aloe-emodin” OR “emodin” OR “aloin” OR “acemannan”) AND (“bioavailability” OR “pharmacokinetics” OR “absorption” OR “metabolism” OR “intestinal permeability” OR “drug delivery” OR “nanoparticles” OR “nanoformulation”); (“*Aloe vera*” OR “aloe-emodin” OR “emodin” OR “aloin” OR “acemannan”) AND (“apoptosis” OR “cell cycle arrest” OR “oxidative stress” OR “reactive oxygen species” OR “ROS” OR “inflammation” OR “angiogenesis” OR “metastasis” OR “pyroptosis” OR “autophagy”) AND (“PI3K” OR “Akt” OR “mTOR” OR “MAPK” OR “ERK” OR “NF-κB” OR “p53” OR “Wnt” OR “β-catenin” OR “JAK” OR “STAT” OR “EMT”); (“*Aloe vera*” OR “aloe-emodin” OR “emodin” OR “aloin” OR “acemannan”) AND (“breast cancer” OR “cervical cancer” OR “colorectal cancer” OR “colon cancer” OR “lung cancer” OR “hepatocellular carcinoma” OR “liver cancer” OR “prostate cancer” OR “melanoma” OR “leukemia” OR “leukaemia” OR “lymphoma” OR “blood cancer”); (“*Aloe vera*” OR “aloe-emodin” OR “emodin” OR “aloin”) AND (“chemotherapy” OR “cisplatin” OR “paclitaxel” OR “doxorubicin” OR “gefitinib” OR “radiotherapy” OR “radiation” OR “radiosensitivity” OR “chemosensitivity” OR “drug resistance” OR “combination therapy” OR “adjuvant therapy”); AND (“*Aloe vera*” OR “aloe-emodin” OR “emodin” OR “aloin” OR “Aloe latex” OR “Aloe gel”) AND (“toxicity” OR “safety” OR “adverse effects” OR “side effects” OR “hepatotoxicity” OR “nephrotoxicity” OR “genotoxicity” OR “mutagenicity” OR “clinical trial”). The literature published during the specific timeframe of 2015–2025 and the peer-reviewed English articles related to *Aloe vera*’s anticancer efficacy, pharmacokinetics, stability, delivery methods, and toxicity were included in this review. Moreover, the non-English language published studies and studies outside this time period were excluded to improve understanding and readability.

## 3. Geographical and Morphological Description

**Figure 1:**
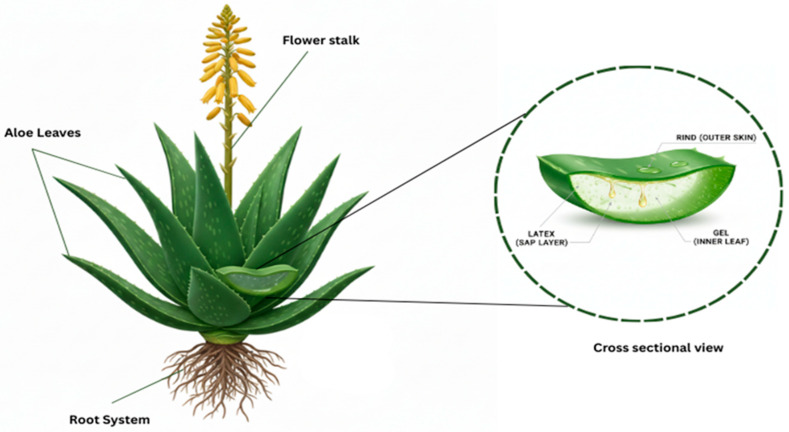
Morphological structure of *Aloe vera* showing the aloe leaves, flower stalk, and root system, along with a cross-sectional view of the leaf highlighting the rind/outer skin, latex sap layer, and inner gel region [5,25,28,29,30,31,32,33,34].

*Aloe vera* (syn. *Aloe barbadensis Mill*.), a perennial, succulent, and innate plant belonging to the *Asphodelaceae* family, is widely distributed in the tropical and subtropical regions of South Africa, Pakistan, India, China, and the Arabian Peninsula [5]. It achieves its maximum growth in the dry and semi-dry conditions during the hot temperatures and low rainfall [28,29,30]. As far as its morphology is concerned, it is usually lined with coarse, fleshy, serrated triangular leaves, with each leaf comprising an outer thick green rind, middle latex layer, and inner clear gel, primarily composed of water and polysaccharides, as described in Figure 1 [25,31]. The leaves grow to large sizes depending on the growing conditions, while the succulent structure makes them resilient to dry climates due to their water storage capability [32,33]. These characteristics and their widespread global distribution exaggerate its commercial and therapeutic significance [34].

## 4. Phytochemical Composition

**Figure 2:**
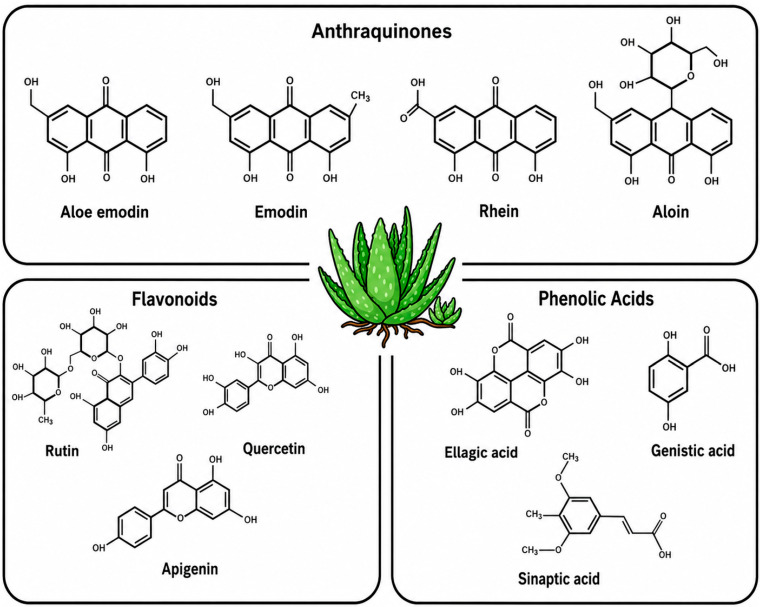
Phytochemicals identified in *Aloe vera*, including anthraquinones (aloe-emodin, emodin, rhein, aloin), flavonoids (quercetin, rutin, apigenin), and phenolic acids (ellagic acid, gentisic acid, sinapic acid) [6,37,38,39,40].

Phytochemicals are the plant-derived bioactive compounds, particularly responsible for the therapeutic and industrial reliability of plants. Various phytochemicals, such as polyphenols, flavonoids, phenolic acids, terpenoids, saponins, alkaloids, carotenoids, and glucosinolates, have been identified, contributing to promoting individual health by attenuating disease severity and progression [35]. The health-promoting and disease-alleviating properties of *Aloe vera* are also due to the phytochemical compounds found in it. For instance, the inner leaf gel contains significant levels of polysaccharides, such as acemannan and glucomannans, which are responsible for immunomodulatory, antioxidant, and wound-healing properties [4,28]. Moreover, flavonoids (quercetin [34.4 mg/100 g], rutin [22.3 mg/100 g], and apigenin [3.3 mg/100 g]), tannins, catechins, phenolic acids (sinapic acid [54 mg/100 g] and gentisic acid [6.0 mg/100 g]) and anthraquinones (aloe-emodin [0.051 mg/g dry powder] and aloin [6.16 mg/g dry powder]) possess antioxidant and anti-inflammatory properties [6,36]. Furthermore, the quantitative phytochemical analyses have identified alkaloids, glycosides, saponins, and other secondary metabolites [37,38]. However, the phytochemical composition is different based on the geographical area, cultivated land, irrigation system, and processing conditions, consequently varying safety as well as biological conditions [36,39]. Collectively, phytochemical diversity supports *Aloe vera* as a powerful natural resource for nutraceutical, cosmetic, pharmaceutical, and food industries [40]; their phytochemical structures are described in Figure 2.

## 5. Bioavailability

Bioavailability is the rate at which drugs, compounds, and nutrients are absorbed and reach the systemic circulation to provide specific health outcomes. Bioavailability is a major concern that needs to be addressed for translating in vitro into in vivo or clinical activity effectively [41]. Compounds, such as aloe-emodin, emodin, aloin, acemannan, flavonoids, and phenolic acids, exhibit promising anticancer activity, but the efficacy of these compounds is dependent on a variety of factors, including absorption, intestinal permeability, metabolism, systemic exposure, tissue distribution, and elimination. Hence, bioavailability should be taken as an important factor in determining the applicability of *Aloe vera* in cancer treatment and not just as another pharmacological property.

Available evidence suggests that anthraquinones present in *Aloe vera* are mainly absorbed in the intestine, with greater absorption in the small intestine than in the stomach or colon. Oral exposure has been shown in rats to result in systemic exposure via intestinal absorption, with anthraquinone absorption to about 67% in the small intestine and 24% in the colon [42]. Aloe-emodin is also found to permeate the intestine in intestinal segments, but the transport into the cells might be altered by intestinal efflux transporters like multidrug resistance-associated protein 2 and P-glycoprotein, which may decrease the cellular accumulation and thereby decrease the bioavailability in systemic circulation [43]. Aloe-emodin and emodin exhibited anticancer activities, such as induction of apoptosis, modulation of ROS, mitochondrial dysfunction, and inhibition of oncogenic signaling, depending on the adequate concentration of aloe-emodin and emodin within the cell and tumor tissue.

However, the oral bioavailability of some anthraquinones found in Aloe is still limited. Aloe-emodin has been reported to be poorly absorbed in the intestine, highly excreted in feces, and has a short half-life after oral administration, which may limit its therapeutic applications following oral administration [44]. Likewise, aloin exhibits poor bioavailability when taken orally; its bioavailability is about 6%, and it has a half-life of about 4 h, meaning it is rapidly excreted from the body. Furthermore, only a small percentage of orally administered aloin is excreted in the urine, indicating a large percentage of aloin is metabolized, transformed by intestinal flora, or excreted in the feces without being completely absorbed. These limitations in the pharmacokinetic parameters may account, in part, for why the results of strong cytotoxic effects seen in cell-culture models are not always directly applicable to animal and human studies.

Another crucial factor that determines the activity of aloe compounds is metabolism. Aloe compounds, during intestinal absorption, can be extensively modified by hydrolysis, glucuronidation, and sulfation. Aloin, aloe-emodin, and aloesin have been reported to be absorbed in part in the glucuronidated and sulfated form, suggesting that the forms and biological activity in circulation may be varied [45]. These conjugated metabolites could help to increase water solubility and elimination, but could also decrease the amount of free active compounds at tumor sites. Hence, future research is needed to not only explore parent compounds, but also to examine the distribution of metabolites in tissues and tumor-specific accumulation.

The polysaccharide part of *Aloe vera*, especially acemannan, poses distinct bioavailability challenges. The molecular weight and the hydrophilic properties of acemannan are also likely to limit the capacity of the small anthraquinones to be absorbed. It might be more relevant for its anticancer action in the context of local intestinal activity, effects on gut-associated immunity, macrophage activation, cytokine regulation, and indirect immune-mediated anticancer effects. So the pharmacokinetic assessment of Aloe polysaccharides must consider plasma concentration, interaction with the gut, activation of immune cells, metabolism by microbiota, and systemic immunomodulatory effect.

Bioavailability and systemic exposure are closely correlated to toxicity. The gel of *Aloe vera* is generally regarded as less hazardous compared to latex; however, some preparations made from the whole leaf or latex from certain species contain anthraquinone, which needs to be carefully evaluated for safety. The chemicals in aloe latex include aloin and aloe-emodin, which can have a laxative effect and may cause gastrointestinal irritation, diarrhea, abdominal cramps, electrolyte imbalance, and dehydration at high doses or with extended use. Antihraquinone-containing preparations should not be taken for too long or in high doses as there may be concerns about hepatotoxicity, nephrotoxicity, genotoxicity, and herb–drug interactions. In oncology, these problems are significant, as patients might already be on chemotherapy, radiotherapy, immunotherapy, targeted therapy, etc. The compounds obtained from aloe can interact with drugs in several ways, such as changing the absorption, activity of efflux transporter proteins, response to oxidative stress, and hepatic metabolism, impacting the efficacy or toxicity of anticancer drugs.

In order to overcome pharmacokinetic limitations, several formulation strategies have been explored or proposed. Selected Aloe compounds, such as aloin, are also suggested to be more bioavailable due to improved intestinal transformation and absorption during fermentation [46]. Solubility, stability, controlled release, intestinal permeability, and tumor targeting can be further enhanced by the use of nanoparticle-based systems, liposomes, polymeric carriers, hydrogels, phytosomes, solid-lipid nanoparticles, and targeted delivery systems. Aloe-based hydrogels could also facilitate local delivery and increase the duration of contact in certain tissues, and nano-formulations could help prevent degradation of unstable compounds and increase the time spent in the target tissue. These strategies; however, necessitate systematic investigation of the following properties: particle size, efficiency of entrapment, release characteristics, pharmacokinetics, bio-distribution, toxicity, and anticancer activity.

## 6. Antioxidant Potential

Oxidative stress, caused by the imbalance of antioxidants and free radicals within the body, is a major health impact, contributing to the progression of various maladies, including cancer, cardiovascular disorders (CVD), liver diseases, and neurodegenerative disorders [47]. The free radicals, particularly reactive oxygen and nitrogen species (ROS and RNS), result in cellular damage, followed by lipid peroxidation and DNA damage during normal biological processes [48]. In this regard, antioxidant-enriched bioactive compounds of medicinal plants, especially *Aloe vera*, play a fundamental role in neutralizing free radicals and reducing oxidative stress. Various studies have evidenced the antioxidant potential of *Aloe vera* and its bioactive constituents [49]. The presence of phenolic compounds (kaempferol and quercetin), flavonoids and anthraquinones, i.e., aloin and aloe-emodin, in *Aloe vera* scavenges free radicals by modulating superoxide dismutase (SOD), catalase (CAT), and glutathione peroxidase (GPx) activity [50,51,52,53,54]. Oxidative stress and chronic inflammation play important roles in cancer initiation, progression, angiogenesis, metastasis, and treatment resistance. *Aloe vera* and its bioactive constituents have shown antioxidant and anti-inflammatory properties through free-radical scavenging, modulation of antioxidant enzymes, and regulation of inflammatory mediators [48]. Regarding cancer, these effects may contribute to reduced DNA damage, suppression of pro-inflammatory signaling, modulation of tumor microenvironment, and enhancement of apoptosis in malignant cells [50]. Moreover, *Aloe vera* compounds may exert a dual redox-modulating effect: they can protect normal cells from oxidative injury while increasing ROS accumulation in cancer cells beyond a toxic threshold, thereby promoting mitochondrial dysfunction, caspase activation, cell-cycle arrest, and apoptosis. This redox-dependent mechanism has been reported in several cancer models, where Aloe-derived compounds inhibited cancer-cell proliferation and enhanced programmed cell death through modulation of oxidative stress-related pathways. Thus, the antioxidant potential of *Aloe vera* provides an important mechanistic basis for its anticancer effects by linking oxidative-stress regulation with apoptosis induction, inflammation suppression, and inhibition of tumor progression. However, antioxidant activity alone is not sufficient to establish anticancer efficacy. Therefore, the following sections focus specifically on experimental and clinical evidence showing how *Aloe vera* and its compounds affect cancer-cell proliferation, apoptosis, metastasis, angiogenesis, immune response, and major oncogenic signaling pathways.

## 7. Anticancer

Cancer, the invasion, proliferation, and malignancy of abnormal cells, is a major cause of mortality worldwide, which is attributed to the modified contributors, such as sedentary lifestyle, excessive consumption of alcohol, tobacco, and processed foods, exposure to a contaminated environment, ultraviolet radiation, tobacco smoking, limited breastfeeding practices, and high body mass index (BMI) [51]. Moreover, this multi-dimensional disease arises at the genetic, epigenetic, metabolic, and transcriptomic levels [52]. Over the last few decades, there has been a significant progress in developing therapeutic strategies, such as radiotherapy, chemotherapy, hormone replacement therapy, immunotherapy, surgery, targeted therapy (tyrosine kinase inhibitors, monoclonal antibodies, HER2 inhibitors, and VEGF inhibitors), bone marrow transplantation, and combination therapy (surgery plus chemotherapy, chemotherapy plus radiotherapy, and targeted therapy plus immunotherapy) to cure cancer [53,54,55]. These regimens aid in attenuating the proliferation of tumor cells, particularly by promoting apoptotic induction, reducing inflammation, regulating immune responses, and inhibiting cancer cell growth [29,56,57,58]. Moreover, nano-formulations [59] and plant-derived phytochemicals [57] have a promising role in cancer management owing to their limited adverse consequences and more therapeutic potential. The anticancer potential of *Aloe vera* has been evidenced by various studies against different cancers, such as hepatocellular carcinoma (HCC), colon cancer, melanoma, lung cancer, breast cancer, and cervical cancer [60].

In a study involving network pharmacology, a database (TCMSP) analysis reported that out of 181 *Aloe vera* possible targets in the human body, 174 are directly linked to cancer. These molecular docking studies showed that anthraquinones, especially quercetin, beta-carotene, and aloe-emodin, target AKT1 and TP53 genes to inhibit cancer growth and regulate apoptosis induction [61]. Moreover, these compounds are also associated with other pathways, including TNF, HIF-1, and p53, to control cell invasion and metastasis [61]. Furthermore, 2D and 3D cultures are used to explore the cancer-suppressing effect of *Aloe vera*, which showed a remarkable decline in cell survival rate of the HCC cell line with the provision of *Aloe vera* [62]. Alongside *Aloe vera* extract, the gel of *Aloe vera* is also gaining a lot of interest in modern studies due to its cytostatic activity against melanoma cells (A375), as a study depicted that *Aloe vera* gel exhibited potent anti-melanoma activity by reducing the colony formation of A375 cells [63]. Beyond its promising effect on melanoma, *Aloe vera* has also shown a remarkable anticancer potential against leukemia. A study was conducted to evaluate the survival rate via MTT assay at different time intervals (24, 48, and 72 h) after the provision of *Aloe vera* to HL-60 leukemia cells. The results revealed that the IC50 of *Aloe vera* was 13.7 µM after 24 h, which was primarily due to the increased expression of Caspase-3 and Caspase-9 [18].

Alongside targeting leukemia, *Aloe vera* has also shown great synergy with other compounds. A study revealed that *Aloe vera* in combination with honey showed potent cytotoxic activity and anti-proliferative effects on in vitro MCF-7 and Walker-256 cells; consequently, it can be used as a co-adjuvant therapy in cancer treatment [64]. In recent years, there has been growing attention towards the role of polysaccharides found in *Aloe vera* in inhibiting colorectal cancer progression as well [65]. Subsequent studies on ethanolic leaf extracts of *Aloe vera* also showed potent anti-proliferative activity against human liver (HepG2), cervical (HeLa), and lung (A549) cancer cell lines [66]. Similar studies revealed that an improvement in radio-sensitivity was observed in HeLa (cervical cancer) and HCC cell lines by Aloe-emodin and anthraquinones [67].

### 7.1. Hepatocellular Carcinoma

HCC is ranked fifth among the most prevalent cancers with the second-highest fatality rate, and the third most common underlying cause of mortality [68]. Hepatocellular carcinoma (HCC) is the primary cancer of the liver that usually progresses through a multistage process, including chronic inflammation, hepatocyte injury, compensatory regeneration, and accumulation of genetic and epigenetic changes. Aloe-emodin, an anthraquinone derived from Aloe species, has been reported to exhibit anti-inflammatory and anticancer activities. Experimental studies indicate that aloe-emodin has the ability to inhibit the progression of hepatocellular carcinoma by negatively regulating the PI3K/AKT signaling pathway, thereby inhibiting cell proliferation and inducing cell death through apoptosis [69]. AE reduced PI3KR1, AKT1, and BCL-2 expression, where BCL-2 acts as an anti-apoptotic protein that normally prevents programmed cell death in cancer cells, and upregulated Fas, Bax, Caspase-3, Caspase-9, PARP, and p53 expression, where Bax promotes mitochondrial membrane permeabilization leading to the activation of apoptotic signaling, to slow down tumor growth and invasion [67,70]. Activation of Caspase-9 subsequently activates Caspase-3, an executioner caspase responsible for DNA fragmentation and apoptotic cell death through cleavage of cellular proteins such as PARP. Modern studies have used 2D and 3D cultures to depict the potent cancer-suppressing effect, which indicated that *Aloe vera* showed a remarkable decline in cell survival rate of HCC cell lines at 12.5 mg/mL [71].

**Figure 3:**
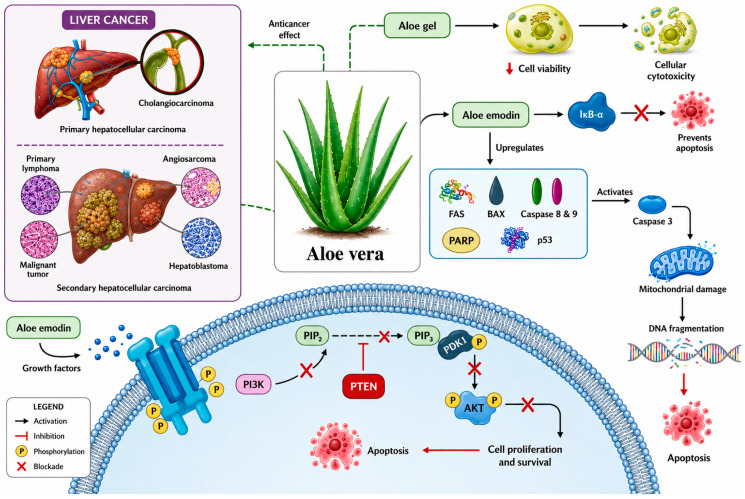
Anticancer mechanisms of *Aloe vera* and its bioactive compound, aloe-emodin, reduce cancer cell viability and induce cytotoxicity by activating apoptotic mediators, such as Fas, PARP, p53, and caspases-3/8/9, while downregulating the anti-apoptotic protein Bcl-2, ultimately leading to mitochondrial damage, DNA fragmentation, and apoptosis [17,67,69,70,71,72,73,74,75,76]. Black arrows indicate activation or progression of the pathway. Red inhibitory bars and red X marks indicate suppression, inhibition, or blockade of signaling events. Red downward arrows indicate reduced cell viability, while red arrows toward apoptosis indicate promotion of apoptotic processes.

The latex extract of *Aloe vera* (200 μg/mL) has anti-carcinogenic potential, which was revealed by the downregulation of BCL-2 and upregulation of Caspase-3, -8, and -9 expression, indicating activation of both intrinsic and extrinsic apoptotic pathways that promote programmed cancer cell death [72]. Caspase-8 is mainly involved in the extrinsic apoptotic pathway that is activated through death receptors such as Fas [73]. Activation of Caspase-8 can directly activate Caspase-3 or amplify the mitochondrial apoptotic pathway through interaction with pro-apoptotic proteins, further promoting apoptosis in cancer cells [74]. Another in vivo study model followed by CCl4 and DEN-induced HCC in BALB/c mice revealed that Emodin isolated from the marine plant, *Halodule uninervis*, induced apoptosis in HepG-2 cells with an IC50 of 49.24 µg/mL [75]. Additionally, the gel fraction strengthened the anticancer profile of *Aloe vera*. Cell viability was significantly decreased in HepG-2 cells when treated with *Aloe vera* gel in a concentration and time-dependent manner, demonstrating the IC50 values of 153 and 80 μg/mL at 24 and 48 h, respectively [17]. Besides its remarkable role in treating cancer, *Aloe vera* also showed a potent hepatoprotective effect against ischemia–reperfusion (I/R) injury and minimized oxidative stress-induced tissue damage [76]. The anticancer mechanism of *Aloe vera* against HCC was illustrated in Figure 3.

### 7.2. Cervical Cancer

Cervical cancer is a major public health concern throughout the world, particularly affecting low- and middle-income countries (LIMCs) [77]. It is ranked second among the common cancers in women after breast cancer, which contributes to ~0.66 million reported new cases and 0.35 million deaths in 2022 [78,79]. Human papillomavirus (HPV) is the most common cause of cervical cancers (~99%), with certain other factors, such as a sedentary lifestyle and poor food practices [80]. The practiced conventional treatments, including radiotherapy, chemotherapy, hormone replacement therapy, and surgery, pose adverse effects, such as muscle wasting, renal and neural damage, hair loss, and drug resistance, which require further adjunct therapies to confront this prevailing concern [81]. Over the last few decades, researchers studied medicinal plants and their compounds in managing cancer progression due to their safe and health-promising attributes [82].

*Aloe vera*, a therapeutic medicinal plant, showed promising anticancer effects in various studies, significantly by targeting both viral infections, oncogenes, and tumor metastasis in cervical cancer cell lines [79]. An in vitro study showed that aloe-emodin found in *Aloe vera* extract attenuated the expression of HPV E6 and E7 proteins, thereby downregulating tumor proliferation, apoptosis induction, and GLUT1 expression on HeLa and SiHa cell lines [83]. Moreover, anthraquinones, the flavonoids found in *Aloe vera*, also revealed great potency in combating cancer cells [84,85]. Ref. [86] in his study found that quercetin not only targeted SERPINE-1 but also influenced E6 protein to reduce the invasion of cervical cancer. Furthermore, quercetin suppressed cancer pathways, such as PI3K/Akt, MAPK, and EMT, to alleviate tumor growth and proliferation. A distinct type of regulated cell death (RCD), also known as pyroptosis, has gained recent attention due to its unique pathway of cell death [87]. Apoptosis leads to cell shrinkage, DNA fragmentation, and ultimately cell death without causing any signs of inflammation [88]; however, pyroptosis results in cell swelling, bubble formation, cell burst, and the release of inflammatory signals to cause inflammation [89]. It has been reported that HeLa cells treated with Aloe-emodin undergo cell death via the modulation of both apoptotic and pyroptotic pathways [86]. Moreover, Aloe-emodin displayed its anti-mutagenic activity by damaging tumor cell mitochondria, which enhances the release of Caspase-3 and -9 enzymes; consequently, the induced cell membrane burst occurs and leads to pyroptosis [90].

**Figure 4:**
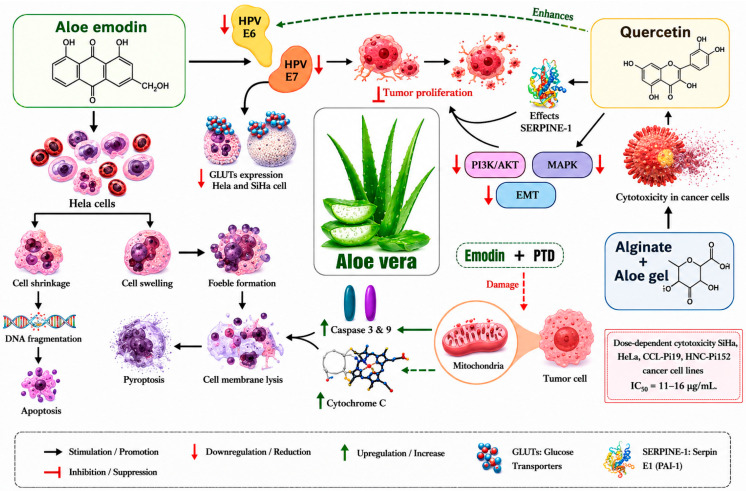
Anticancer mechanisms of *Aloe vera* against cervical cancer. Phytochemicals, such as aloe-emodin, quercetin, and emodin, inhibit HPV E6/E7 oncogenic proteins, suppress PI3K/Akt and MAPK signaling pathways, and reduce tumor proliferation. Moreover, these compounds induce apoptosis and pyroptosis via caspase activation, mitochondrial damage, and DNA fragmentation. Black arrows indicate stimulation or pathway progression. Red inhibitory symbols indicate inhibition or suppression, red downward arrows indicate downregulation or reduction, and green upward arrows indicate upregulation or increase [78,79,83,84,85,86,87,88,89,90,91].

Aloe-emodin, in conjunction with chemotherapeutic drugs, improves the effectiveness of drugs and makes cancer cells more sensitive to drugs [90]. Another in vitro study supplemented emodin in combination with photodynamic therapy (PDT) to evaluate its cytotoxicity against cervical cancer cell lines, which showed that emodin causes mitochondrial membrane damage via the release of cytochrome-c and the activation of caspase cascade (caspase-9 and caspase-3), suggesting the synergistic anticancer potential of emodin and PDT [85]. The gel structure of *Aloe vera* also showed some promising anticancer potential. Aloe gel, when combined with alginate, showed promising cytotoxicity by reducing cervical cancer cell viability. Additionally, the formation of Aloe-alginate hydrogel also improved the stability, adhesion, and delivery system, thereby allowing the compounds to be released slowly to prolong their therapeutic effect [78]. Furthermore, nanoparticle-based delivery systems, particularly silver nanoparticles, prepared using *Aloe vera* gel, were evaluated against cervical cancer progression. The dose-dependent cytotoxicity was observed against SiHa, HeLa, CC1-PI19, and HNCF-PI52 cancer cell lines with an IC_50_ value of 11–16 µg/mL [91]. The anti-proliferative mechanism of *Aloe vera* against cervical cancer is illustrated in Figure 4.

### 7.3. Colorectal Cancer

**Figure 5:**
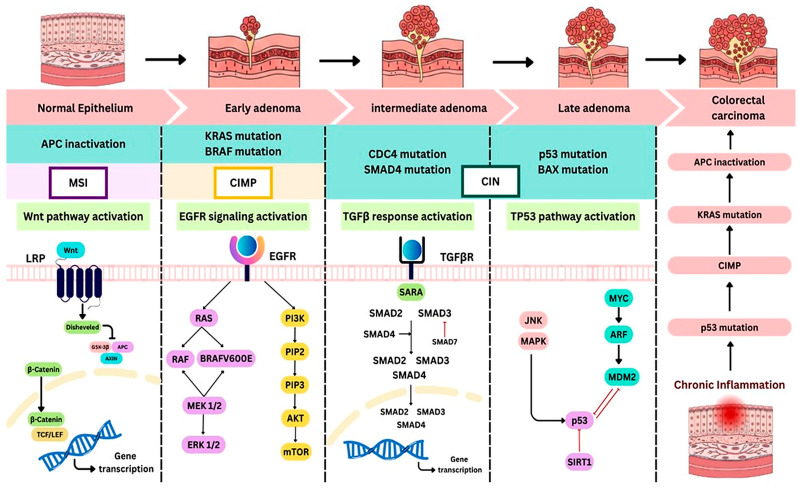
Adenoma–carcinoma sequence from normal epithelium to colorectal carcinoma. Tumor initiation occurs through APC inactivation and aberrant Wnt/β-catenin signaling. Early adenoma development is driven by KRAS or BRAF mutations activating EGFR–MAPK and PI3K–AKT–mTOR pathways, often associated with CIMP. Progression involves disruption of TGF-β/SMAD signaling and increased chromosomal instability (CIN). Late-stage transformation is characterized by TP53 and BAX mutations, leading to impaired cell cycle control and apoptosis, ultimately resulting in colorectal carcinoma [92,93,94,95,96]. Black arrows indicate activation, signaling, transcriptional regulation, or progression between events. Red inhibitory lines indicate suppression or negative regulation. Dashed vertical lines separate major stages of colorectal tumor progression. The pink ribbon represents histopathological progression from normal epithelium to colorectal carcinoma, while the teal band denotes key genetic alterations acquired during tumorigenesis.

Colorectal cancer (CRC) is among the third most prevalent cancer worldwide, with an estimated 2 million newly diagnosed cases and ~1 million deaths recorded annually [92]. Over the past three decades, there has been a significant increase in the incidence rate of colorectal cancer, such as 17% increase in South Asia and 25.9% in the Western Pacific region [86,93]. It has been documented that poor lifestyle practices, including alcohol consumption, smoking, obesity, sedentary lifestyle, and stress, alongside genetic predisposition (mutation in the APC gene) to radiation exposure, are the major contributors responsible for the development and progression of CRC [94]. The pathogenesis of CRC involves several molecular pathways, particularly CIN (chromosomal instability), the most common pathway involved in APC gene mutation; MSI (microsatellite instability), another mechanism that occurs when the DNA repair system fails; and the CpG island methylator phenotype (CIMP), responsible for inhibiting tumor suppressor genes due to abnormal DNA methylation [95]. Moreover, the activation of the PI3K signaling pathway leads to reduced apoptosis, uncontrolled metabolism, and excessive cell growth [96]. Figure 5 describes the pathogenesis of CRC comprehensively.

The management of CRC through medicinal plants, particularly *Aloe vera*, has attained significant attention due to their therapeutic attributes [97]. Emodin, a potent anthraquinone found in *Aloe vera*, is well known for its natural anti-inflammatory properties and is involved in preventing CRC progression. It has been reported by [98] in his study that supplementing emodin (40 and 80 mg/kg) to the respective genetic model of intestinal cancer (Apc^Min/+^) and a chemically induced model of CRC [azoxymethane/dextran sodium sulfate (AOM/DSS)] thrice a week enhanced the immune system’s ability to fight cancer by targeting macrophages, thereby decreasing M2-type (pro-tumor) macrophages and increased M1 (anti-tumor) macrophages. Moreover, emodin also suppressed P2X7 protein, which is responsible for inducing inflammation and making the body even more immunocompromised. Similarly, *Aloe vera* has also shown dose-dependent results in reducing CRC proliferation by inhibiting MMP-9 (Matrix Metalloproteinase-9) enzyme, responsible for cancer cell invasion and metastasis [99]. Another study evaluated the effect of *Aloe vera* on chemically induced CRC. Phthalates, the most common plastic chemical found in bottles, packaging, and cosmetics, when exposed to colorectal cells, caused higher cancer cell migration, viability, abnormal glycosylation, and increased stemness. Ref. [100] extracted *Aloe vera* polysaccharides, such as A50 and I50, containing acemannan, and evaluated their potential on CRC. The results showed that administering *Aloe vera* polysaccharides reversed the abnormal glycosylation, reduced cell viability and migration, thereby revealing their potent cytotoxic and anticancer activity. Table 1 illustrates the preclinical studies associated with the anticancer potential of *Aloe vera* against CRC.

### 7.4. Lung Cancer

Lung cancer contributes to the cancer-associated mortalities in both men and women globally, with ~0.2 new cases and ~0.13 million demises are recorded by the American Cancer Society annually [108,109]. Lung cancer is categorized into non-small-cell lung carcinoma (NSCLC), accounting for ~80–85% of all lung tumors, and small-cell lung carcinoma (SCLC), accounting for the remaining ~15–20% of lung cancer [110,111,112]. Its pathogenesis is linked to the heavy metals’ exposure (cadmium, mercury, and arsenic), environmental pollution, inappropriate lifestyle (smoking, alcohol consumption, and tobacco intake), and dietary practices [113]. Although effective therapeutic strategies in the form of chemotherapy and radiotherapy exist, the clinical results of lung cancer management are still disappointing, with low morbidity and mortality rates alongside persistent toxicity concerns [114]. Moreover, other side effects, such as drug resistance during long-term clinical manipulation, are another major problem, which demand a safer and more effective alternative, e.g., plant-based strategies, as they have been associated with delaying progression and decreasing risk of cancer [111]. The impact of aloe-emodin was studied on the development and progression of non-small cell lung cancer (NSCLC). Ref. [113] evaluated aloe-emodin on lung cancer cell lines (A549 and H460), and the results demonstrated that 20 and 40 μM of AE slowed down and ceased cancer cell proliferation by overloading them with oxidative stress and generating ROS, along with activating caspase enzymes, resulting in apoptosis and autophagy. Moreover, Ref. [111] conducted a comparative study to investigate the effect of glycosylated aloe-emodin (AE3G) and the aglycone form of aloe-emodin on A549 human lung cancer cells. The 5–50 μM AE3G has shown relatively better results in targeting the mitochondria of cancer cells, thereby damaging their function. Additionally, it also triggered apoptosis by activating pro-apoptotic protein (Bax), decreasing anti-apoptotic proteins, and blocking Akt and MEK pathways. Another anti-proliferative mechanism was observed when *Aloe vera* extract was combined with royal jelly and applied to non-small cell lung cancer cells (Koprulu). Synergistically, they enhanced the cytotoxicity of cancer cells, induced apoptosis, and reduced toxicity. Moreover, royal jelly also revealed anti-inflammatory and antioxidant activity.

Ref. [87] in his study developed a new *Aloe vera* gel extract, AVBEC (*Aloe vera* barbadensis extract C), without using any organic solvents or freeze drying to preserve *Aloe vera*’s natural bioactive compounds and avoid structural degradation of certain sensitive compounds, thereby making it more biocompatible and safer for clinical use. The onco-protective potential of this extract was evaluated on both small cell lung carcinoma (NCL-H524) and non-small cell lung carcinoma (NCL-H1975) cell lines. Results demonstrated that AVBEC generated ROS in the cancer cells, consequently damaging their mitochondria and leading to the release of pro-apoptotic proteins (AMPK, Bax, and cytochrome-C), thereby inducing apoptosis and attenuating their proliferation. Another study co-administered emodin (0, 10, 20, 40, 80, 120 μM) with paclitaxel (0, 2, 4, 8, 16, 32 μM) showed increased cytotoxicity in the A549 cell line by inhibiting cancer cell invasion and migration, thereby making them more sensitive to treatment and reducing their drug resistance [110]. Table 2 demonstrates the anticancer activity of *Aloe vera* and its compounds against lung cancer.

### 7.5. Blood Cancer

Leukemia is characterized as a heterogeneous group of hematopoietic malignancies followed by uncontrolled proliferation and differentiation of blood stem cells, ultimately resulting in overproduction of immature cells called ‘blasts’ in the bloodstream [123,124]. Leukemia starts when the hematopoietic stem cells or progenitor cells undergo genetic modifications and become mutated, resulting in clonal proliferation and tumor heterogeneity [125,126]. Leukemia is linked to white blood cells; therefore, any blood-forming cell in the bone marrow can also transform and develop into a leukemic cell [127]. Alongside genetic modifications, several other factors, such as compromised immunity, radiation exposure, and carcinogenic chemicals, such as benzene, are involved in leukemia development [128,129]. Benzene is a harmful environmental pollutant that deteriorates bone marrow, causes abnormal growth of white blood cells, and consequently develops anemia among individuals. Ref. [130] induced leukemia in male Wistar rats using benzene and observed genotoxicity, oxidative stress, bone marrow damage, abnormal-shaped RBCs (poikilocytosis), and increased WBC count (leukocytosis). *Aloe vera*, well known for its antioxidant and therapeutic properties, was supplemented in this model. The gel structure of *Aloe vera* demonstrated geno-protective effects and showed hematological protection by restoring Hb, RBC, and WBC, and reducing the number of blast cells. Moreover, an increase in arylesterase enzyme activity, which is responsible for breaking down harmful oxidants and restoring thiol proteins, was also observed, highlighting *Aloe vera*’s role as a potent antioxidant [119]. *Aloe vera* extract examined on HL-60 cell line showed anti-proliferative effect along with increasing caspase-3 and -9 levels to modulate apoptosis and tumor cell suppression [18]. Another study evaluated the onco-protective effect of *Aloe vera* gel extract (AVG) and aloe-emodin on K562 (human chronic myeloid leukemia cells), HL-60 (human promyelocytic leukemia cells), and P3HR-1 (human Burkitt’s lymphoma) cell lines. It has been observed that aloe-emodin demonstrated strong cytotoxic activity by inducing apoptosis in K562 and P3HR-1 cells via caspase dependant pathway with an IC_50_ value of 60.98 ± 0.90 and 28.06 ± 1.69 µM, respectively [131]. However, *Aloe vera* gel extract showed stronger cytotoxicity by inducing apoptosis through a caspase-independent pathway, suggesting *Aloe vera*’s role as a promising natural anticancer agent [122]. The other studies related to the effect of *Aloe vera* and its bioactive compounds on leukemia are demonstrated in Table 3.

### 7.6. Breast Cancer

Breast cancer continues to pose a major health threat, affecting ~2.2 million individuals worldwide annually, with the mortality rate of 15 per 100,000 cases in women and ~0.34 per 100,000 cases in men [133,134,135]. Its complex development and diverse symptoms require the need for effective treatment and prevention. The emergence of plant-based therapeutic strategies in recent years has gained considerable attention for their safe and more effective results with minimal side effects [119]. Studies on *Aloe vera* have demonstrated strong cytotoxicity and anti-proliferative effect against breast cancer cell lines. Ref. [135] investigated the role of *Aloe vera* leaf extract against MCF-7 breast cancer cell lines. Results showed significant cytotoxic activity against MCF-7 cells with an IC_50_ value of 23 µg/mL in contrast to the IC_50_ value of 332 µg/mL in NIH-3T3 (normal cells), demonstrating low toxicity towards normal cells [126]. In addition to the leaf extract, the gel structure of *Aloe vera* also depicted great potency in combating breast cancer cells due to the possession of anthraquinones, polysaccharides, phenolic compounds, and flavonoids, which are responsible for inducing apoptosis, scavenging free radicals, and modulating mitochondrial metabolism [136]. Another study evaluated the effect of *Aloe vera* crude extract on MCF-7 breast cancer cells. A dose and time-dependent relationship was observed in reducing MCF-7 cell line viability by inducing apoptosis, followed by upregulating Bax and p21 protein and downregulating cyclin D1, CYP1A1, and CYP1A2 enzymes [137]. Additionally, molecular docking results further demonstrated that the bioactive compounds found in *Aloe vera*, especially riboflavin, daidzin, and aloin, interact and incorporate into the DNA structure of the cancer cells, thereby blocking DNA replication and tumor cell growth. Moreover, riboflavin showed the strongest DNA binding affinity than the standard drug daunorubicin in MM/PBSA analysis, supporting *Aloe vera*’s anticancer potential [138]. Table 4 enumerates the cytotoxic potential of *Aloe vera* against breast cancer cell lines.

### 7.7. Prostate Cancer

Prostate cancer is one of the most common cancers in men and is the 2nd leading cause of cancer-related mortality in men worldwide [143,144,145]. It has been reported that prostate cancer causes ~7.3% of all cancer cases in men and ~3.8% of all cancer-related mortalities in 2020 [146]. Despite advances in research, the exact cause of prostate cancer is still not known [147]. However, certain major risk factors include impaired androgen metabolism, dietary modifications, ethnicity, mutated oncogenes, and age, as it mainly affects older men in the latter half of their lives [148]. Moreover, studies have shown that the interaction between certain molecular pathways, such as androgen receptor (AR), NF-κB, and Wnt/β-catenin pathways, plays a significant role in tumor progression and proliferation [149,150]. The androgen receptor is a protein that regulates prostate growth and is responsible for the development and progression of prostate cancer. Abnormal modulation of this protein can lead to increased prostate cell proliferation, thereby increasing the risk of mutation and cancer formation [148]. The metastasis of this cancer usually affects 90% bone, 46% lungs, 25% liver, and 13% adrenals [132]. Conventional treatments, such as surgery, chemotherapy, and radiation therapy, have significant adverse effects that can reduce the quality of life and are often limited to advanced metastatic cases as well. Moreover, resistance to chemotherapy is another major problem [145]. Therefore, compounds extracted from natural sources are much safer and more effective alternatives, which show significant efficacy against cancer cells [151]. Regarding these challenges, the exploration of *Aloe vera* as a natural therapeutic agent has gained considerable attention due to its diverse bioactive constituents. A recent cell culture-based study has demonstrated that *Aloe vera* extract showed potent cytotoxic activity against DU145 prostate cancer cells [142]. Results showed that *Aloe vera* extract increased ROS levels inside cancer cells, leading to apoptosis induction via oxidative stress and cellular damage [152]. Moreover, *Aloe vera* extract induced apoptosis by damaging the mitochondria, leading to leakage of cytochrome-c, activating caspases [5,11,12], thereby causing DNA fragmentation and nuclear condensation [153]. Furthermore, molecular docking results confirmed that *Aloe vera* extract can strongly bind to Wnt2 and β-catenin to suppress signaling pathways, which helps prostate cancer cells to grow, survive, and resist drugs [153,154]. Moreover, *Aloe vera* acts as an anti-proliferative and tumor-suppressing agent. Aloe-emodin, a potent anthraquinone, is well known for its cytotoxicity, which activates CD82 protein for suppressing tumor metastasis in the PC3 cell line. Prostate cancer cells are often linked with PI3K activation and PTEN loss, activating Akt and mTOR2 pathways to uncontrolled cell growth and survival. CD82 protein counters this by downregulating Rac1, which interferes with PI3K, Akt, and mTOR signaling pathways to stop cell metastasis [144]. Furthermore, aloe-emodin also showed efficacious onco-protective activity against LNCaP cells, androgen-dependent prostate cancer cells. Aloe-emodin suppresses the androgen receptor, resulting in proteasomal degradation of the androgen receptor (AR). It also reduced the mRNA expression of PSA (prostate-specific antigen), KLK2 (kallikrein-related peptidase 2), and TMPRSS2 (Transmembrane serine protease 2) genes, which are responsible for prostate cancer progression [153].

**Figure 6:**
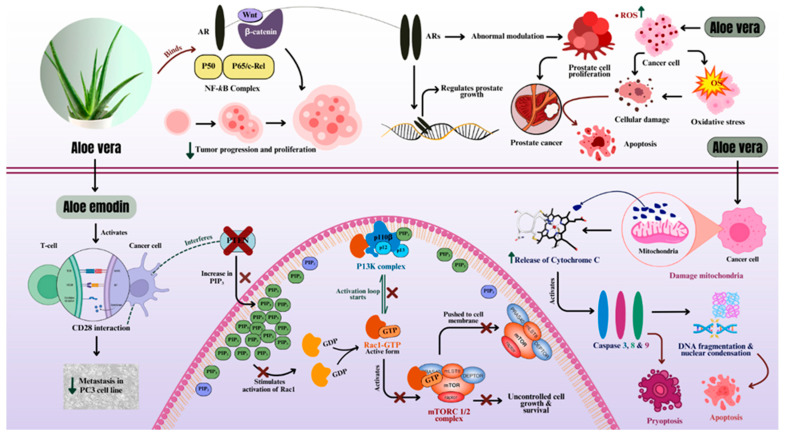
Anticancer mechanisms of *Aloe vera* and aloe-emodin in prostate cancer. *Aloe vera*-derived compounds modulate androgen receptor (AR) signaling and inhibit tumor progression by regulating NF-κB, Wnt/β-catenin, and PI3K/Akt/mTOR signaling pathways. Moreover, Aloe-emodin induces oxidative stress, mitochondrial dysfunction, and cytochrome-c release, leading to activation of caspases-3, -8, and -9 [142,144,146,149,150,152,153,154,155,156].

Beyond its influence on AR-dependent and AR-independent prostate cancer cells, the cytotoxic efficacy of aloe-emodin also depends on the P53 gene, which acts as a guardian and protects the cell from becoming cancerous. Another study investigated aloe-emodin’s effect on prostate cancer cell lines, having different p53 status with LNCaP, DU-145, and PC3 having normal, mutated, and no p53 at all, respectively. The results demonstrated that LNCaP cells were the most sensitive while PC-3 cells were the least affected, concluding that aloe-emodin works best when p53 is functional [144]. Moreover, aloe-emodin also activated caspase-8 and -9, thereby damaging cancerous cells by triggering apoptosis and pyroptosis [155]. Another important investigation focused on *Aloe vera* gel extract on tumour-suppressing and antioxidant properties against PC3 cells. The cytotoxicity was measured using the MTT assay, which demonstrated that *Aloe vera* gel extract reduced cancer cell viability in a dose-dependent manner, with stronger cytotoxic activity at 400 µg/mL [146]. Moreover, *Aloe vera* gel extract also showed potent radical scavenging activity with maximum potential at 200 µg/mL [156]. This shows that *Aloe vera* not only suppresses cancer cell growth but also protects against oxidative stress. Beyond the gel and the leaf extracts, even the flowers of *Aloe vera* have drawn attention for their potential anticancer effects. Ref. [9] in his study investigated the cytotoxic effect of ethanolic extracts from the flowers of aloe species, namely *Aloe vera*, Aloe rubroviolacea, and Aloe sabaea, against PC3 cell lines. The results showed that *Aloe vera* flower extract had the strongest cytotoxic activity, significantly inhibiting the growth and proliferation of cancer cells. Figure 6 presents the anti-metastatic potential of *Aloe vera* against prostate cancer.

## 8. Other Cancers

*Aloe vera* reveals the protective effect against other types of cancer as well, which are demonstrated in Table 5. Aloe-emodin exhibited significant cytotoxicity towards T24 bladder cancer cells through inhibition of cell viability, G_2_/M cell-cycle arrest, upregulation of p53, p21, Bax, and caspase-3, and downregulation of anti-apoptotic protein Bcl-2, indicating activation of the Fas/APO-1-mediated apoptotic pathway [154]. In gastric cancer models, aloin inhibited the proliferation and migration of human gastric cancer cells (HGC-27 or BGC-823) by down-modulating ROS production and blocking Akt/mTOR/Stat3/NF-κB signaling pathways as well as cyclin D1, N-cadherin, MMP-2, and MMP-9, and upmodulating E-cadherin [157]. In addition, the leaf and gel extracts of *Aloe vera* also showed cytotoxic activity in HCT116 colon cancer cells at the dose of 75–150 μg/mL by reducing cell viability [158], and the crude extract of *Aloe vera* caused a decrease in cell viability in HL-60 leukemia and MCF-7 breast cancer cells [159]. Aloe-emodin arrested the cells in G2 phase and promoted apoptosis, decreased cell proliferation, cell migration, and cell invasion in melanoma cells, and suppressed tumor growth by regulating the Wnt/β-catenin pathway [160]. Furthermore, the protein extract of *Aloe vera* had inhibitory activity on the proliferation of human neuroblastoma cell lines, such as IMR-32, TGW, CHP-126, and NBL [161]. Aloe-emodin also inhibited the viability of SCC15 oral cancer cells (IC50 ~ 60.90 µM) and promoted apoptosis by activation of caspase-9 and caspase-3 [162]. In skin cancer models, aloe-emodin resulted in better inhibition of the viability of cutaneous squamous carcinoma SCC-25 and melanoma MUG-Mel2 cells in comparison with emodin, especially when used in combination with photodynamic therapy [163]. In addition, methanolic *Aloe vera* leaf extract demonstrated antiproliferative activity against HeLaS3 uterine cancer cells [164], and aloe-emodin inhibited proliferation, migration, colony formation, and tumor growth in nasopharyngeal carcinoma cells through down-regulation of lncRNA D63785 and suppression of the PI3K/Akt/mTOR pathway [165]. In summary, these results suggest that *Aloe vera* can be a multi-targeted anticancer agent in various cancer models, primarily by inducing apoptosis, arresting cell cycle, blocking migration and invasion, modulating oxidative stress, and regulating major oncogenic signaling pathways.

## 9. Synergistic Effect of *Aloe vera* with Other Plants and Phytochemicals

Synergistic activity is demonstrated by the combination of two or more drugs, compounds, or plants to improve the potency and efficacy of each other for better health outcomes. *Aloe vera* reveals significant synergistic interactions when combined with other phytochemical and pharmaceutical agents. A recent study showed that *Aloe vera* and polysaccharides, such as allantoin and xanthan, formed hydrogels, in combination, had stronger antibacterial and wound healing activity as compared to *Aloe vera* used alone [12]. Similarly, in vitro and in vivo experiments have shown that bioactive compounds (aloe-emodin, rhein, and emodin) of *Aloe vera* interact closely with EDTA and enhance the sensitivity of resistant Gram-negative bacteria to polymyxins, thereby increasing the bactericidal effect [130]. Another study found that the use of processed Aloe elements, such as flower and gel, contributed to the development of extracellular matrix signaling and wound healing response, signifying the synergistic effect of Aloe on tissue repair pathways [166]. Preclinical pharmacological studies also revealed that combining *Aloe vera* with metformin resulted in a significant decline of collagen accumulated in the lung interstitium and alveolar walls [167]. In a nutshell, *Aloe vera,* alongside other botanicals, excipients, or drugs, produces stronger antimicrobial, wound healing, and anti-fibrotic potential. Some of the synergistic interactions are demonstrated in Table 6.

## 10. *Aloe vera* and Aloe-Derived Compounds with Chemotherapy and Radiotherapy

The modern healthcare systems have developed strategies, particularly radiotherapy and chemotherapy, for the treatment of cancers; however, these treatments have adverse health consequences, and most of the individuals develop resistance to these treatments. To cope with these effects, studies were conducted to combine *Aloe vera* with the chemotherapeutic and radio-therapeutic procedures, so that cancer-preventive properties and individual life span should be improved. The table illustrates the combination effect of *Aloe vera* and its compounds with chemotherapy and radiotherapy in cancer management. Table 7 reveals the combination effect of *Aloe vera* and its compounds with chemotherapy and radiotherapy.

## 11. Human Study and Clinical Evidence

Various in vitro and in vivo studies have demonstrated the anticancer properties of *Aloe vera* and its bioactive constituents; however, there has been limited human evidence, with the majority focusing on supportive cancer care rather than direct tumor inhibitory effects. The majority of clinical studies currently available have focused on the use of Aloe preparations to prevent or treat the effects of chemotherapy or radiotherapy, especially oral mucositis, stomatitis, and radiation-induced dermatitis. Thus, there is potential evidence in the current literature to support the use of *Aloe vera* in oncology, but it is not yet a proven standalone therapy for cancer. Ref. [170] found that the mouthwash using *Aloe vera* solution was as effective as benzydamine solution in decreasing the severity of mucositis in patients undergoing radiotherapy for head and neck cancer and had limited side effects. Moreover, in chemotherapy-induced oral toxicity, Ref. [171] performed a randomized controlled trial in patients suffering from lymphoma and leukemia, and concluded that *Aloe vera* solution had a marked effect in reducing the severity of stomatitis, pain, and improving nutritional status and patient satisfaction in the chemotherapy-induced patients. Similarly, Ref. [172] found that *Aloe vera* topical gel was effective in the prevention of chemotherapy-induced oral mucositis in children with acute lymphoblastic leukemia (ALL). In another randomized clinical trial [173], the effect of *Aloe vera* and Olive oil was compared with sodium bicarbonate in a group of children with grade 3–4 chemically induced oral mucositis, and both treatments were found to be effective in comparison with the sodium bicarbonate group.

But it is not all good news in the clinic. Ref. [174] performed a double-blind randomized phase III trial to assess a blend of natural agents—comprising propolis, *Aloe vera*, calendula, and chamomile, which was compared to a placebo in patients with head-and-neck cancer who were undergoing chemoradiotherapy. There was no significant decrease in grade 3 acute mucositis, indicating that a multi-agent formulation containing Aloe does not necessarily prevent mucositis during chemoradiotherapy. In recent times, Ref. [175] demonstrated that long-term use of *Aloe vera* mouthwash reduced the severity of oral mucositis in weeks 4–6 in patients with head and neck cancer. A systematic review and meta-analysis, published additionally, indicated that *Aloe vera* might be beneficial for patients with severe oral mucositis undergoing radiotherapy or chemotherapy. Furthermore, in a network meta-analysis, Ref. [176] found that *Aloe vera* juice showed high ranking among the mouthwashes used in preventing oral mucositis in patients receiving radiotherapy or chemotherapy, but emphasized the need for further large randomized trials. There have also been clinical reports of radiation-induced dermatitis. In a randomized trial, Ref. [177] used topical application of *Aloe vera* gel during breast cancer therapy with adjuvant radiotherapy, with no significant effect observed on the prevalence and severity of radiation dermatitis. However, in head-and-neck cancer, patients treated with concurrent chemoradiotherapy (CCRT) who were treated with topical application of *Aloe vera* gel experienced some reduction in the moderate to severe radiation dermatitis outcomes, such as erythema, moist desquamation, and burning sensation, whereas the overall radiation-induced skin reaction score showed no significant difference between the groups [178]. In a systematic review and cumulative analysis of randomized controlled trials, Ref. [179] found that prophylactic use of *Aloe vera* was significantly effective in decreasing the overall risk of RID, especially grade 2 and grade 3 RIDs, highlighting the need for larger and well-designed trials. Furthermore, Ref. [180] observed benefits for the use of the cream with Aloe in the prophylaxis and early treatment of radiation dermatitis in patients with breast cancer in the mild stage. Conclusively, human studies have shown that *Aloe vera* has potential protective effects on the reduction of some of the side effects of chemotherapy and radiotherapy, specifically, oral mucositis and radiation dermatitis, in the period of 2015–2025. However, these studies have yet to demonstrate anticancer activity in humans. The existing translational evidence is still preliminary, as most clinical studies only assessed symptom control but not tumor response, progression-free survival, overall survival, or cancer-related biomarkers. Standardized preparations of *Aloe vera*, standardized doses, clinically relevant tumor cohorts, PK monitoring, safety assessment, evaluation of herb–drug interaction, and clinically relevant endpoints (tumor response, QoL, treatment-related toxicity, PFS, and OS) should be considered for future clinical trials.

## 12. Limitations and Future Perspectives

Although the bioactivity of *Aloe vera* and its compounds has shown potential anticancer effects in several experimental models, some important gaps are still missing in the current literature. The majority of evidence is from in vitro studies in cancer cell lines, and there are relatively few in vivo studies and minimal clinical trials available. Thus, the clinical significance of these results is not yet clear. The studies with cancer cell lines can be helpful to identify molecular mechanisms, but difficult to evaluate the complexity, bioavailability, metabolism, immune interactions, tumor microenvironment, and systemic toxicity of human tumors. The inconsistent standardization of the preparations of *Aloe vera* is another limitation of this review. Previous literature has utilized whole-leaf extract, gel extract, latex extract, and crude extract, isolated anthraquinones, polysaccharides, and nano-formulations, and their phytochemical composition significantly differs based on plant species, geographical location, growing conditions, ripeness, extraction solvent, processing method, and storage conditions. Therefore, comparisons across studies are impossible, and there is limited reproducibility. Future studies should be well-designed, clearly defining the extract evaluation, reporting the amount of the tested bioactive components (aloe-emodin, emodin, aloin, and acemannan), and standardizing the dose.

Another limitation is their bioavailability. Some components of *Aloe vera*, such as anthraquinones, may have poor oral absorption, high metabolism, and low systemic availability following oral administration. Hence, pharmacokinetic studies are required to study absorption, distribution, metabolism, elimination, plasma half-life, tissue distribution, and active metabolites. Nanoparticles, hydrogels, liposomes, and targeted carriers are advanced delivery systems that should be further investigated to improve stability, solubility, tumor targeting, and therapeutic efficacy. Moreover, the anticancer activity of *Aloe vera* should be further validated. Recently, studies have indicated that some compounds in *Aloe vera* modulate the pathways of apoptosis, pyroptosis, oxidative stress, angiogenesis, metastasis, immune response, as well as signaling pathways including PI3K/Akt, MAPK, NF-κB, Wnt/β-catenin, p53, and HIF-1α. But numerous studies report pathway modulation without direct molecular targets. Advanced techniques such as transcriptomics, proteomics, metabolomics, molecular docking, gene silencing, CRISPR-based validation, and pathway-specific inhibitors should be used in future investigations to validate causal mechanisms. Assessment of safety and toxicity is also crucial. *Aloe vera* is generally regarded as a natural therapeutic agent; however, safety cannot be guaranteed by being natural. The effects of long-term use, high-dosage supplements, latex use with anthraquinones, drug–herb interactions, genotoxicity, hepatotoxicity, nephrotoxicity, gastrointestinal irritation, and the effects of these on normal proliferating cells need to be systematically examined. Because *Aloe vera* may affect the metabolism, therapeutic effects, or toxicity of one or more drugs, this is especially important when *Aloe vera* is used along with chemotherapy, radiotherapy, immunotherapy, or targeted therapy. Further studies should be focused on well-designed preclinical and clinical studies. For in vivo studies, tumor-bearing animal models should be employed, multiple doses, positive controls, toxicity endpoints, and follow-up should be utilized. Randomized controlled trials with standardized formulations of *Aloe vera*, standardized doses, treatment duration, pharmacokinetic study, safety evaluation, quality of life assessment, and tumor response assays are suggested for clinical studies. The studies are required to establish the safety and efficacy of the translation of *Aloe vera* and its bioactive compounds from experimental models of cancer to evidence-based adjunctive cancer therapy.

## 13. Conclusions

*Aloe vera* has emerged as a biologically versatile medicinal plant with substantial relevance to cancer research due to its rich phytochemical profile and multi-targeted mechanism of action. The studies summarized in this review demonstrate that both crude extracts and isolated bioactive constituents of *Aloe vera*, particularly anthraquinones (aloe-emodin, emodin, and aloin), along with polysaccharides (acemannan), exert anticancer effects across a wide spectrum of malignancies, including hepatocellular, cervical, colorectal, lung, breast, prostate, and hematological cancers. These effects are consistently observed in both in vitro and in vivo models and are mediated through modulation of key cellular processes, such as apoptosis, pyroptosis, oxidative stress regulation, immune response activation, angiogenesis inhibition, and suppression of metastasis. Mechanistically, *Aloe vera* and its associated compounds influence multiple oncogenic signaling pathways, including PI3K/Akt, MAPK, NF-κB, Wnt/β-catenin, p53, and HIF-1α, underscoring their capacity to target cancer progression at genetic, metabolic, and epigenetic levels. Moreover, direct cytotoxic effects on tumor cells, *Aloe vera* demonstrates selective toxicity, often sparing normal cells, and exhibits synergistic interactions with chemotherapeutic agents, photodynamic therapy, and nanotechnology-based delivery systems. These attributes position *Aloe vera* as a promising candidate for adjunctive cancer therapy aimed at enhancing efficacy while minimizing adverse effects.

## Figures and Tables

**Table 1:** Preclinical studies investigating the anticancer activity of Aloe compounds in colorectal cancer.

Study Type	Colorectal Cell Line	Extract/Compound	Dose	Key Outcomes	Reference
In vitro	DLD-1, WiDr	Aloe-emodin	0.30–0.37 mM	AE induced apoptosis, release of AIF and cytochrome-c, ↑ caspase-3, ↓ and casein kinase II	[101]
In vitro and in vivo	LS1034	Emodin	50 μM	Emodin induced ROS, mitochondrial dysfunction, ↑ Bax, ↓ Bcl-2, ↑ apoptosis, and ↓ xenograft tumor growth.	[102]
In vitro and in vivo	HT-29	Aloin (AL)	10–40 µM	↓tumor angiogenesis, growth via ↓ STAT3 activation, ↓ VEGF/angiogenic signaling, and tumor size	[103]
In vitro	HCT116, SW480, SNU-C2A, SNU-C5	Emodin	25 and 50 µM	↓ proliferation, FASN expression, and impaired colon cancer cell growth	[104]
In vitro	SW620, HT-29	Aloe-emodin (AE)	10, 20, and 40 μM	↑ ER stress, PERK, eIF2α, CHOP signaling, mitochondrial apoptosis and ↓ SW620 and HT-29 cells migration	[105]
In vitro	DLD-1 and COLO-20	Emodin	10, 20, 40, and 80 μM	↓ viability, ↑ apoptosis via caspase activation, and altered Bcl-2 family expression; selective cytotoxicity toward cancer	[106]
In vitro	HT-29	Aloe arborescens leaf extracts, aloe-emodin, aloin	A. arborescens Extracts: 0.1–1 mg/mL Aloin: 5 µM Aloe-emodin: 5 µM	↓ cell viability, dose-dependent inhibition of HT-29	[107]

↑ = increase; ↓ = decrease.

**Table 2:** In vitro and in vivo studies evaluating the anticancer effects of *Aloe vera*-derived compounds on human lung cancer cell lines.

Study Type	Lung Cell Line	Extract/Compound	Dose	Key Outcomes	Reference
In vitro	CH27	Aloe-emodin	40 µM	Induced apoptosis, DNA fragmentation, caspase activation, and mitochondrial pathways	[115]
In vitro	H460	Aloe-emodin	40 µM	Induced apoptosis via modulation of PKA, PKC, Bcl-2, caspase-3, and p38	[20]
In vitro (photodynamic study)	H460	Aloe-emodin	20 μM	Induced photocytotoxicity/anoikis in H460 cells via mitochondrial changes and caspase pathways	[116]
In vitro (mechanistic)	Human NSCLC cell lines, A549, H1703	Emodin	25–200 μM	Induced cytotoxicity via ERK1/2 inactivation, down-regulation of Rad51 and ERCC1, and upregulation of thymidine phosphorylase (TP)	[117]
In vitro	A549, H1650	Emodin	2–10 µM	Enhanced gefitinib cytotoxicity, reduced p-ERK1/2 and Rad51	[118]
In vitro	A549	Emodin	10–80 µM	Inhibited A549 growth, reduced colony formation, and induced apoptosis	[119]
In vitro	A549	Emodin	60 µM	Inhibited A549 proliferation, reduced colony formation, and altered apoptosis-related gene expression	[119]
In vitro	A549	Aloin	100, 200, 300, and 400 µM	Induced apoptosis in A549 via ROS, MAPK signaling, and p53 phosphorylation	[120]
In vitro + In vivo	A549	Barbaloin	50 and 100 µM	inhibited A549 proliferation, induced G2/M arrest, increased caspase activation, reduced migration, and suppressed tumor growth and hepatic metastasis	[121]
In vitro	A549	Whole ethanolic *Aloe vera* leaf extract	3.9, 7.8, 15.6, 31.2, 62.5, 125, 250, and 500 µg/mL	Inhibited proliferation of the A549 cell line	[122]

**Table 3:** Effects of *Aloe vera* and related bioactive compounds on leukemia and lymphoma models.

Study Type	Cancer Cell Line	Extract/Compound	Concentration	Key Outcomes	Citation
In vitro	HL-60 human acute myeloid leukemia cells	*Aloe vera* crude extract	IC_50_: 1 mg/mL after 24 h	↓ cell viability, apoptotic modifications, TUNEL-positive cells, no reported cytotoxicity	[132]
In vitro	HL-60 human leukemia cells	*Aloe vera* extract	IC_50_: 13.1 µM	↑ cytotoxicity against HL-60 cells, ↑ Caspase-3 and Caspase-9 gene-expression	[17]
In vitro	K562 chronic myelogenous leukemia and P3HR-1 Burkitt’s lymphoma cells	Aloe-emodin	IC_50_: 60.9 µM for K562; 28 µM for P3HR-1	↑ Cytotoxicity in K562 and P3HR-1 cells, ↑ apoptotic induction	[122]
In vitro	K562 chronic myelogenous leukemia cells	*Aloe vera* gel	IC_50_: 243.2 µg/mL	↑ Cytotoxicity in K562 cells, ↑ apoptotic cells reached 46.7% in K562 cells	[122]
In vivo	Benzene-induced leukemia in male Wistar rats	*Aloe vera* gel	150 mg/kg for 7 days	Restored hematological parameters toward normal, ↓ anisocytosis, poikilocytosis, blast-cell occurrence, bone-marrow dysplasia, and micronucleus frequency	[119]

↑ = increase; ↓ = decrease.

**Table 4:** Molecular and cytotoxic effects of *Aloe vera*-derived phytochemicals in breast cancer models.

Study Type	Breast Cell Line	Extract/Compound	Dose/Concentration	Key Outcomes	Reference
In vitro	MCF-7, MDA-MB-453	Aloe-emodin and emodin	Emodin: 25–100 µM; Aloe-emodin: 6–100 µM	Dose-dependent suppression of MCF-7 proliferation via down-regulation of ERα protein stability, aloe-emodin promoted HSP90/ERα dissociation and ubiquitination.	[139]
In vitro	MCF-7	Methanolic extract of *Aloe vera* leaf	0–500 µg/mL; IC_50_ 74.33 µg/mL	Dose-dependent cytotoxic effect on MCF-7 cells; time- and concentration-dependent growth inhibition.	[140]
In vitro	MCF-7	Crude extract of *Aloe vera* (ACE)	1–60%	ACE caused significant viability loss; when combined with cisplatin, synergy was observed. Down-regulation of cyclin D1 and up-regulation of Bax/p21.	[141]
In vitro	MCF-10AT, MCF-7	Aloe-emodin	0–100 µM	Inhibition of proliferation and induction of apoptosis by up-regulating miR-15a and miR-16-1, which suppresses Bcl-2.	[142]

**Table 5:** Anticancer potential of *Aloe vera* against various cancerous cell lines.

Cancer Type and Cell Line	Agent	Main Results	Author
Bladder cancer	Aloe-emodin	↓ viability of T24 cells, ↑ G_2_/M arrest, ↑ p53, p21, Bax expression, ↓ Bcl-2, ↑ caspase-3 activation via Fas/APO-1 portal	[154]
Gastric cancer	Aloin	↓ ROS production, Akt/mTOR/Stat3/NF-κB signaling, tumor proliferation and migration of HGC-27 and BGC-823 cells, ↓ cyclin D1, N-cadherin, MMP-2/9; ↑ E-cadherin	[157]
Colon cancer	*Aloe vera* leaf/gel extracts	cytotoxic effect of extracts on HCT116 colon cells via ↓ cell viability	[158]
Leukemia (HL-60) and breast cancer (MCF-7) cell lines	Crude *Aloe vera* extract	cytotoxicity in HL-60 and MCF-7 cells (leukemia and breast) via apoptosis induction	[159]
Skin cancer (A375 and SK-MEL-28 cells)	Aloe-emodin	inhibited proliferation, migration, and invasion of melanoma cells; induced G2 phase cell cycle arrest and apoptosis; suppressed tumor growth, an effect tied to the Wnt/β-catenin pathway	[160]
Human neuroblastoma cell lines (IMR-32, TGW, CHP-126, NBL)	*Aloe vera* protein extract	suppressed proliferation	[161]
Oral cancer (SCC15 cells)	Aloe-emodin	inhibited SCC15 cell viability (IC_50_ ~60.90 µM), induced apoptosis via increased caspase-9 and caspase-3.	[162]
Skin cancer (cutaneous squamous carcinoma SCC-25 and melanoma MUG-Mel2)	Aloe-emodin vs. emodin + photodynamic therapy	Aloe-emodin is more effective than emodin in reducing the viability of skin cancer cells, especially under PDT	[163]
Uterine cancer (HeLaS3)	Methanolic extract of *Aloe vera* leaves	Anti-proliferative effect on HeLaS3 cells	[164]
Nasopharyngeal carcinoma (CNE1 and C666-1)	aloe-emodin	inhibited proliferation, migration, colony formation; downregulated lncRNA D63785, suppressed PI3K/Akt/mTOR signaling; reduced tumor growth	[165]

↓ = decrease; ↑ = increase.

**Table 6:** Synergistic interaction of *Aloe vera* with other plants, drugs, and phytochemicals.

Property	*Aloe vera* Combination	Main Findings (Synergistic Role)	Reference
Antibacterial activity	*Aloe vera* gel + Cymbopogon (lemongrass) essential oil	↑antibacterial activity against multiple skin infections and pathogens	[168]
	*Aloe vera* + Manuka honey/Citrus honey + Mint essential oil + Indian costus	Enhanced antimicrobial and antibiofilm activity against pathogens (MRSA, P. aeruginosa)	[169]
	*Aloe vera* extract + Antibiotics (Ciprofloxacine)	*Aloe vera* methanolic extract showed enhanced antibacterial activity alongside standard antibiotics against *S. aureus*, *E. coli*, and *Klebsiella*	[170]
Wound healing	*Aloe vera* + Turmeric (*Curcuma longa*)	Accelerated wound contraction, faster healing, and enhanced tissue regeneration	[171]
	*Aloe vera* + Curcumin	Better wound healing properties (earlier tissue regeneration and collagen deposition)	[172]
Dermatological effect	*Aloe vera* + Trimethylglycine	upregulated Aquaporin-3 expression and improved skin hydration	[173]

**Table 7:** Combination of *Aloe vera* and Aloe-derived compounds with chemotherapy and radiotherapy.

Study Type	Cancer Model	Extract/Compounds	Conventional Therapy Combined	Dose	Proposed Mechanism	Citation
In vitro	MCF-7 and HeLa cell lines	*Aloe vera* crude extract	Cisplatin	Low-dose extract combined with cisplatin	↑ Bax and p21, ↓ cyclin D1, CYP1A1, and CYP1A2, ↑ apoptosis induction	[130]
In vitro	A549 and MCF-7 cancer cells	*Aloe vera* extract	Paclitaxel	24 µg/mL *Aloe vera* extract + 5 µM paclitaxel	↑ Apoptosis induction and anti-migratory activity	[166]
In vitro	A549 and MCF-7 cancer cells	*Aloe vera* extract	Paclitaxel	24 µg/mL *Aloe vera* extract + 5 µM paclitaxel	↓ ECAR, ↑ BAX, ↑ apoptosis regulation, cell-cycle, ribosomal, and translational pathways	[166]
In vitro	A549 and H460 cancer cells	Emodin	Cisplatin	Low-dose emodin combined with cisplatin	↓ P-glycoprotein, ↑ caspase-mediated apoptosis	[168]
In vitro and in vivo	Gefitinib-resistant non-small cell lung cancer cells	Aloe-emodin	Gefitinib	Aloe-emodin combined with gefitinib	Reversal of EMT, ↓ PI3K/Akt/Twist1 signaling	[169]

↓ = decrease; ↑ = increase.

## Data Availability

Data will be available upon request from the corresponding author.
